# Prospective associations of sleep duration and screen time with transition from overweight/obesity to normal BMI in U.S. adolescents

**DOI:** 10.1038/s41366-025-01980-6

**Published:** 2025-12-23

**Authors:** Abubakr A. Al-Shoaibi, Christiane K. Helmer, Kyle T. Ganson, Alexander Testa, Jason M. Lavender, Erin E. Dooley, Kelley Pettee Gabriel, Orsolya Kiss, Fiona C. Baker, Jason M. Nagata

**Affiliations:** 1 Department of Pediatrics, University of California, San Francisco, CA USA; 2 Factor-Inwentash Faculty of Social Work, University of Toronto, Toronto, ON Canada; 3 Department of Management, Policy and Community Health, University of Texas Health Science Centre at Houston, Houston, TX USA; 4 Military Cardiovascular Outcomes Research Program (MiCOR), Department of Medicine, Uniformed Services University of the Health Sciences, Bethesda, MD USA; 5 The Metis Foundation, San Antonio, TX USA; 6 Department of Epidemiology, University of Alabama at Birmingham, Birmingham, AL USA; 7 Center for Health Sciences, SRI International, Menlo Park, CA USA

**Keywords:** Risk factors, Epidemiology

## Abstract

**Background:**

Shorter sleep duration and longer screen time are established risk factors for adolescent obesity. However, the extent to which these behaviors are prospectively associated with the transition back from overweight/obesity to a healthy status remains unclear. We examined whether sleep duration and screen time among adolescents with overweight/obesity are associated with the likelihood of transitioning to a normal body mass index (BMI).

**Methods:**

We used data from 3498 U.S. adolescents aged 9–11 years with overweight/obesity (45.1% female), from the Adolescent Brain Cognitive Development (ABCD) Study. Cox proportional hazards models examined the prospective associations of parent-reported sleep duration (9–11, 8–9, 7–8, and <7 h/day) and screen time (hours/day) with a shift from overweight/obesity (BMI percentile ≥85) to a normal (BMI percentile <85) accounting for key covariates including pubertal status.

**Results:**

Over a median 657 days of follow-up, 643 (18.4%) adolescents transitioned from overweight/obesity to a normal BMI percentile. Compared with those sleeping 9–11 h, adolescents sleeping 7–8 h were less likely to transition to a normal BMI percentile (hazard ratio [HR]: 0.60, 95% CI 0.44, 0.82), with significant dose-response trend (p for trend = 0.003). The association remained significant in sex-stratified analyses for both females (HR: 0.55, 95% CI 0.30, 0.98) and males (HR: 0.59, 95% CI 0.41, 0.86), with similar significant trend in both groups (p for trend <0.05). Higher screen time was not associated with transitioning to a normal BMI overall (HR: 0.99, 95% CI 0.96, 1.02) or by sex (females, HR: 1.00, 95% CI 0.95, 1.05; males, HR: 0.99, 95% CI 0.95, 1.02).

**Conclusion:**

Short sleep duration was prospectively associated with a lower likelihood of transitioning to a normal BMI among adolescents with overweight/obesity. This association warrants further investigation as a potential intervention target.

## Introduction

The prevalence of overweight and obesity is increasing among youth in the United States (U.S.). In 2021, approximately 15.1 million children and adolescents (aged 5–14 years) had overweight/obesity, and this number is projected to increase by an additional 3.33 million by 2050 [1]. Overweight/obesity during adolescence is often linked to weight-related stigma, which has been associated with adverse mental health outcomes, including higher anxiety and depression symptoms [2]. In addition to psychological consequences, overweight/obesity is associated with a greater risk for adverse cardiometabolic outcomes such as diabetes and high blood pressure [3]. Furthermore, overweight/obesity among children and adolescents aged 4–19 years is associated with excess medical care costs [4].

Prior research has suggested a strong association between short sleep duration and the risk of overweight and obesity [5, 6]. A systematic review and meta-analysis of longitudinal studies suggested that short sleep duration was associated with subsequent overweight/obesity in children and adolescents [6]. Moreover, a dose-response meta-analysis of prospective studies reported that short sleep duration was associated with the risk of obesity in children and adolescents, particularly those within 3–13 years of age [5]. Short sleep duration is commonly associated with changes in levels of hormones that control hunger and satiety, potentially promoting higher energy intake [7]. Short sleep duration is also associated with reduced physical activity, which may further contribute to the risk of weight gain over time [8].

Several studies have linked screen time to obesity among adolescents. For example, a systematic review of cross-sectional studies found that high screen time was associated with a 1.27 times higher likelihood of having overweight/obesity among adolescents [9]. Another systematic review and meta-analysis of cross-sectional and longitudinal studies found that screen time ≥2 h/day compared to <2 h/day was associated with a greater risk of having overweight/obesity [10]. Potential mechanisms that may underlie observed associations between screen time and obesity include high food intake, low physical activity, and insufficient sleep [11].

Prior studies have explored how factors such as sleep and screen time may contribute to the risk of obesity among adolescents [5, 6, 9, 10]. However, the role of these factors in the transition from overweight/obesity to normal body mass index (BMI) among adolescents remains unclear. Randomised controlled trials in adolescents show that behavior-change lifestyle interventions targeting sleep and screen time as part of multicomponent programs addressing multiple behaviors [12–14] can produce modest reductions in BMI [12, 14, 15], but prospective, naturalistic, population-based studies on adolescents are lacking. Understanding if these factors may be associated with reductions in BMI could inform the development or refinement of effective behavioral and/or public health interventions for overweight/obesity among adolescents.

During adolescence, a transition from overweight/obesity to a normal BMI may reflect either weight loss or weight maintenance concurrent with increases in height due to normal growth and pubertal development [16]. Therefore, examining BMI transitions during this period may provide insight into both biological and behavioral processes associated with changes in BMI.

This study aimed to address gaps in the existing literature by examining the prospective associations of sleep duration and screen time with the transition from overweight/obesity to normal BMI percentile among adolescents in the U.S. We hypothesized that adolescents with overweight/obesity who had shorter sleep duration or higher screen time would be less likely to shift to a normal BMI percentile over time compared to those with higher sleep duration or lower screen time.

## Materials and methods

### Participants

We analysed longitudinal data from the Adolescent Brain Cognitive Development (ABCD) Study, a prospective cohort study of brain development and child health in the U.S. The ABCD study recruited 11,875 adolescents aged 9–10 years from 21 sites at baseline (2016–2018) across the U.S. Further details about the study sample, recruitment, protocol, and measures have been reported elsewhere [17]. Due to the COVID-19 pandemic, a large portion of participants completed the Year 2 assessment remotely because of social distancing requirements and the cancellation of non-essential research activities, and therefore, did not complete anthropometric measurements. For this analysis, we used pooled data from the ABCD Study at baseline, Year 1, and Year 2. Participants who did not meet the criteria for overweight/obesity (BMI percentile ≥85th) at either baseline or Year 1, those who transitioned from overweight/obesity to underweight (BMI percentile <5th), and those with missing data for BMI, sleep duration, and screen time were excluded, resulting in a final sample of 3,498 participants. Institutional review board approval was obtained from the University of California, San Diego (160091), and the respective IRBs of each study site. Written assent was obtained from participants, and written informed consent was obtained from their caregivers.

### Follow-up

This analysis utilized data from the first visit (baseline or Year 1) that an ABCD participant met BMI criteria for overweight/obesity, and who later transitioned to a normal BMI at either Year 1 or 2, or who reached the Year 2 assessment without transitioning. In total, 1882 participants were followed from the ABCD Study baseline to Year 2, 1186 were followed from the ABCD Study baseline to Year 1, and 430 were followed from Year 1 to Year 2.

### Outcome

#### Body mass index percentile

Body weight and height were measured three times by trained research assistants, and the average of the three readings was used to calculate BMI as body weight (kg) divided by the square of height (m²). Age- and sex-specific BMI percentiles were calculated according to the Centers for Disease Control and Prevention (CDC) guidelines. We used BMI percentiles rather than BMI z-scores because our endpoint was the transition between the CDC’s clinical weight categories, and since those categories are defined by percentile cut-offs, this was the most direct way to measure this change [18]. Percentile categories also avoid compression of BMI z-scores at very high BMI values [19]. BMI percentile outliers were excluded following the criteria from the CDC growth charts: BMI z-scores ≤-4 standard deviations (SDs) or ≥8 SDs [20]. BMI percentile was grouped into two categories: normal (BMI <85th percentile) and overweight/obesity ( ≥85th percentile).

### Exposures

#### Sleep duration

Sleep duration was estimated using a single item from the Sleep Disturbance Scale for Children (SDSC). Parents or caregivers responded to the question, “How many hours of sleep does your child get on most nights in the past six months?”, which provided an estimate of sleep duration using five response options (9–11 h, 8–9 h, 7–8 h, 5–7 h, and <5 h per day). Given the small number of participants with sleep duration <5 h, this category was merged with the 5–7 h group to form a single category ( <7 h).

#### Screen time

The ABCD Youth Screen Time Survey was completed by adolescents, who reported their own average daily time spent on different screen modalities, including viewing/streaming TV shows or movies, watching/streaming videos (e.g., YouTube), playing video games, texting, video chatting (e.g., Skype, FaceTime), and using social media (e.g., Facebook, Instagram, Twitter). The average daily screen time in hours was computed using the following formula: [(weekday average x 5) + (weekend average x 2)]/7. Self-reported smartphone screen use has shown a significant moderate correlation (*r* = 0.49) with screen time measured objectively using a passive-sensing smartphone app [21]. Screen time was also categorized into three categories: 0–4 h/day (low; reference), >4–8 h/day (medium), and >8 h/day (high) [22–24].

### Covariates

Covariates were selected based on prior research linking sociodemographic [25, 26], psychosocial [27, 28], developmental [3], and behavioral [29] factors to BMI changes in adolescents. Covariates were obtained from the first assessment (baseline or Year 1) at which each participant met the criteria for overweight or obesity (BMI percentile ≥85th). Adverse childhood experiences (ACEs) were assessed only at the ABCD baseline, so baseline ACEs were used for all participants. Covariates included age (years), sex (female or male), race/ethnicity (Asian, Black, Latino/Hispanic, Native American, White, and other), household income (U.S. dollars, six categories: less than $25,000, $25,000 to $49,999, $50,000 to $74,999, $75,000 to $99,999, $100,000 to $199,999, and $200,000 or greater), highest parent education (high school or less vs. college or more), study site, physical activity, depressive symptoms, ACEs score, and self-reported puberty status.

#### Physical activity

Physical activity was obtained from the Sports Activity Involvement Questionnaire (SAI-Q), which was administered to parents/caregivers at baseline, assessing lifetime and past year involvement in 23 different sports, extracurricular activities (e.g., music), and other hobbies (e.g., drawing). Age-specific metabolic equivalent of task (MET) value was assigned to each sport and extracurricular activity based on the 2011 Youth Compendium of Physical Activities [30, 31]. The mean hours per week for each sport and extracurricular activity endorsed was calculated using the following formula: [(duration per session * days per week * months per year*4.33)/ (52*60)]. MET-h/week scores were computed by multiplying weekly participation hours by MET values and summing across activities. Activities with MET values below the moderate intensity threshold of 3.0 METs (e.g., playing music, drawing) were excluded from the summary estimate. Physical activity was categorized into 2 groups (meeting vs. not meeting guideline of ≥21 MET-h/week) [30–32].

#### Depressive symptoms

Depressive symptoms were assessed using the depression subscale of the Child Behavior Checklist (CBCL) DSM-Oriented Scales, completed by parents/caregivers at Baseline [33]. Items were scored from 0 (‘not true’) to 2 (‘very true/often true’), and responses were summed to create a depressive symptoms score.

#### Adverse childhood experiences (ACEs)

The ACEs score was calculated using adolescent and parent responses from the baseline survey, assessing nine of ten ACEs from the original CDC-Kaiser surveys [34]. These included physical abuse, sexual abuse, emotional neglect, physical neglect, household substance use, divorce/separation, household mental illness, violence, and criminal justice involvement. A cumulative score was computed by summing ‘yes’ responses from either the child or caregiver, indicating lifetime exposure to any ACE [35]. ACEs scores were grouped into 5 categories: 0, 1, 2, 3, and 4 + , based on prior research linking higher ACE exposure to greater health risks [35–37].

#### Puberty status

Pubertal development is associated with increases in lean body mass and adiposity driven by gonadal sex steroid production [3, 38]. Adolescents self-reported their pubertal stage using the Pubertal Development Scale (PDS) [39]. Puberty status was grouped into 5 categories (prepubertal, early pubertal, mid-pubertal, late pubertal, and post-pubertal).

### Statistical analysis

Missing data for covariates were imputed using multiple imputation by chained equations (MICE) in Stata. Imputed variables included puberty status (*N* = 655, 18.7%), household income (*N* = 304, 8.7%), parental education (*N* = 3, 0.1%), and depressive symptoms (*N* = 1, 0.03%). We generated 10 imputed datasets. The imputation model included all covariates, exposures, and the outcome, and estimates were pooled following Rubin’s rules.

Differences in characteristics at baseline or Year 1 between females and males were tested by *t*-test for continuous variables and chi-squared test for categorical variables. We used the Cox proportional hazards regression model to assess the prospective independent associations of screen time (continuous and categorical) and sleep duration (ordinal) with the transition from overweight/obesity to normal BMI percentile. Statistical models were adjusted for age, sex, race/ethnicity, household income, highest parent education, study site, physical activity, depressive symptoms, ACEs score, and puberty status. To examine dose-response relationships, sleep duration was included as a continuous variable in the Cox regression model to test for a linear trend. The p-value for the trend was derived from the Wald test. To assess potential effect modification on the multiplicative scale, we included interaction terms between sleep duration and physical activity (meeting vs not meeting guidelines), screen time and physical activity, and sleep duration and screen time. We also tested interactions between sex and each main exposure and conducted sex‑stratified analyses by fitting separate Cox models for females and males with the same covariates, as in the primary model. The statistical significance of all interaction terms was determined using two-sided p-values from the Wald test.

A complete case sensitivity analysis was conducted, including participants without missing data. All analyses were performed using Stata 18 (College Station, TX), and sampling weights based on the American Community Survey were applied [40]. A threshold of *p* <0.05 was considered significant.

## Results

Out of 3,498 adolescents (aged 9–11 years at first assessment), 45.1% were female, 57.6% identified as non-White, and 64.7% were from families with household incomes less than $75,000. A total of 18.3% reported having 4 or more ACEs, and 71.0% did not meet recommended daily physical activity guidelines. Parents reported that 43.9% of children typically slept 8–9 h/day, 34.9% slept 9–11 h, 16.4% slept 7–8 h, and 4.9% slept less than 7 h (Table 1).

**Table 1:** Characteristics of the participants at first assessment when meeting BMI criteria for obesity/overweight (Baseline or Year 1), Adolescent Brain Cognitive Development (ABCD) Study.

	All	Female	Male	*p*-value
	*N* = 3498	*N* = 1578	*N* = 1920	
Sociodemographic characteristics	Mean (SD) / %	Mean (SD) / %	Mean (SD) / %	
Age (years), mean (SD)	10.0 ( ± 0.7)	10.0 ( ± 0.7)	10.1 ( ± 0.7)	**0.008**
Race/ethnicity (%)				**<0.001**
Asian	3.9%	3.0%	4.7%	
Black	21.5%	24.9%	18.6%	
Latino / Hispanic	26.4%	24.2%	28.3%	
Native American	3.8%	4.7%	2.95%	
White	42.4%	44.9%	43.8%	
Other	1.9%	2.3%	1.6%	
Household income (%)				**0.037**
Less than $25,000	24.4%	23.9%	24.7%	
$25,000 through $49,999	23.5%	26.1%	21.3%	
$50,000 through $74,999	16.8%	17.0%	16.6%	
$75,000 through $99,999	13.4%	13.1%	13.7	
$100,000 through $199,999	17.9%	16.1%	19.2%	
$200,000 and greater	4.1%	3.7%	4.5%	
Parent with college education or more (%)	75.4%	73.9%	76.6%	0.098
Sleep duration (h/day)				0.613
9–11	34.9%	33.7%	35.8%	
8–9	43.9%	45.0%	43.0%	
7–8	16.4%	16.2%	16.5%	
<7	4.9%	5.0%	4.7%	
Total screen time, (h/day), n (%)				**<0.001**
Low (0–4)	1965 (53.7%)	962 (59.0%)	998 (49.2%)	
Medium ( >4–8)	1091 (31.9%)	444 (28.5%)	643 (34.7%)	
High ( >8)	452 (14.4%)	172 (12.4%)	279 (16.1%)	
Total screen time, (h/day), mean (SD)	4.6 ( ± 3.2)	4.3 ( ± 3.2)	4.9 ( ± 3.2)	**<0.001**
Physical activity (MET h/week)				**<0.001**
<21	71.0%	75.1%	67.6%	
≥21	28.9%	24.9%	32.4%	
Adverse childhood experiences (Number of ACEs)				0.388
0	20.9%	21.5%	20.4%	
1	26.9%	25.4%	28.1%	
2	20.1%	20.8%	19.5%	
3	13.8%	14.4%	13.2%	
4+	18.3%	17.8%	18.8%	
Depressive symptoms (raw score)	1.4 ( ± 2.2)	1.3 ( ± 2.1)	1.6 ( ± 2.2)	**0.005**
Pubertal status				**<0.001**
Prepubertal	20.7%	14.6%	26.0%	
Early pubertal	36.6%	26.6%	45.2%	
Mid-pubertal	36.7%	50.0%	25.4%	
Late pubertal	5.2%	7.8%	3.0%	
Post-pubertal	0.7%	1.0%	0.4%	

Sampling weights were applied to yield representative estimates based on the American Community Survey from the U.S. Census.

*SD* standard deviation, *N* is the number of participants in each category.

Bold indicates significant results.

During a median of 657 days of follow-up, 643 (18.4%) adolescents transitioned from overweight/obesity to normal BMI percentile. Table 2 shows adjusted hazard ratios (HRs) and 95% confidence intervals (CIs) for the results of Cox proportional hazards models. Compared to participants with a sleep duration of 9–11 h, those sleeping 7–8 h were less likely to transition to normal BMI percentile (HR: 0.60, 95% CI 0.44, 0.82). A significant trend was observed across sleep duration categories (*p* for trend = 0.003), suggesting a dose-response relationship in which shorter sleep durations were associated with a lower likelihood of transitioning to a normal BMI percentile. Similarly, sleep duration of 7–8 h was less likely to transition to a normal BMI percentile both in females (HR: 0.55, 95% CI 0.30, 0.98) and in males (HR: 0.59, 95% CI 0.41, 0.86). Significant dose-response relationships were observed in both females (*p* for trend = 0.018) and males (*p* for trend = 0.017). Sleep duration interaction by sex was not statistically significant (*p* for interaction = 0.598).

**Table 2:** Prospective associations between sleep duration and progression from overweight/obesity to normal BMI percentile in the Adolescent Brain Cognitive Development (ABCD) Study.

	Overall	Female	Male
	*N* = 3498	*N* = 1578	*N* = 1920
	HR (95% CI)	HR (95% CI)	HR (95% CI)
Sleep duration (h/day)			
9–11	Ref	Ref	Ref
8–9	0.83 (0.68, 1.02)	0.81 (0.56, 1.16)	0.81 (0.64, 1.05)
7–8	**0.60 (0.44, 0.82)**^a^	**0.55 (0.30, 0.98)**^a^	**0.59 (0.41, 0.86)**^a^
<7	0.77 (0.45, 1.30)	0.45 (0.13, 1.56)	0.79 (0.42, 1.49)
P-trend	**0.003**	**0.018**	**0.017**
Total screen time (categorical) (h/day)			
0–4	Ref	Ref	Ref
>4–8	0.99 (0.80, 1.22)	1.05 (0.72, 1.56)	0.98 (0.76, 1.27)
>8	1.00 (0.74, 1.35)	1.23 (0.71, 2.15)	0.91 (0.63, 1.32)
Total screen time (continuous) (h/day)	0.99 (0.96, 1.02)	1.00 (0.95, 1.05)	0.99 (0.95, 1.02)

Model adjusted age, sex, race/ethnicity, household income, highest parent education, study site, physical activity, depressive symptoms, ACEs score, and puberty status.

*HR* Hazards Ratio

^a^Bold font indicates significant results.

There was no significant association between screen time and the likelihood of transitioning to a normal BMI percentile, whether modeled as a continuous variable (overall, HR: 0.99, 95% CI 0.96, 1.02; females, HR: 1.00, 95% CI 0.95, 1.05; males, HR: 0.99, 95% CI 0.95, 1.02) or as categorical variable. This null association was consistent in both females and males when analysed separately (Table 2). Screen time by sex interaction was not statistically significant (p for interaction = 0.767). No evidence of effect modification was detected for sleep duration × physical activity; screen time × physical activity; and sleep duration × screen time (all p for interaction >0.05).

In complete-case sensitivity analyses, associations of sleep duration with the transition to a normal BMI were attenuated but remained significant overall (HR: 0.70, 95% CI 0.50, 0.97) and in males (HR: 0.69, 95% CI 0.48, 0.99), likely reflecting reduced power due to the smaller sample size; however, effect estimates remained directionally consistent with the primary analysis (Table 3).

**Table 3:** Sensitivity analyses: hazard ratios and the 95% confidence intervals for the prospective associations of sleep duration and screen time with the transition from overweight/obesity to normal BMI percentile using only participants with complete data in the Adolescent Brain Cognitive Development (ABCD) Study.

	Overall	Females	Males
	*N* = 2598	*N* = 1040	*N* = 1558
	HR (95% CI)	HR (95% CI)	HR (95% CI)
Sleep duration (h/day)			
9–11	Ref	Ref	Ref
8–9	0.89 (0.68, 1.15)	0.81 (0.57, 1.15)	0.91 (0.64, 1.29)
7–8	**0.70 (0.50, 0.97)**^**a**^	0.65 (0.28, 1.55)	**0.69 (0.48, 0.99)**^**a**^
<7	0.85 (0.48, 1.52)	0.26 (0.03, 1.93)	0.94 (0.41, 2.07)
P-trend	0.091	0.097	0.265
Total screen time (categorical) (h/day)			
0–4	Ref	Ref	Ref
>4–8	1.03 (0.80, 1.34)	1.11 (0.66, 1.89)	1.04 (0.77, 1.40)
>8	0.93 (0.67, 1.28)	0.73 (0.19, 2.85)	0.97 (0.65, 1.46)
Total screen time (continuous) (h/day)	0.98 (0.96, 1.02)	0.94 (0.83, 1.06)	1.00 (0.97, 1.04)

Model adjusted age, sex, race/ethnicity, household income, highest parent education, study site, physical activity, depressive symptoms, ACEs score, and puberty status.

*HR* Hazards Ratio.

^a^Bold font indicates significant results.

## Discussion

In this prospective cohort study of adolescents with overweight/obesity, which aimed to examine the associations of sleep durations and screen time with the transition to normal BMI percentile, we found that 2855 (81.6%) continued to have a BMI percentile >85th across the course of their participation. Adolescents with short sleep duration (7–8 h per day) were less likely to transition to normal BMI percentile, both in females and in males. Screen time was not associated with transition to normal BMI percentile. Furthermore, this study observed a dose-response relationship, with shorter sleep durations associated with a lower likelihood of transitioning to a normal BMI percentile in both females and males. Our findings add to the literature by suggesting that shorter sleep duration may play a role in the persistence of overweight/obesity in youth, highlighting a factor that may impact the likelihood of transition from overweight/obesity to normal BMI during adolescent development.

This study highlights that most adolescents with overweight/obesity are unlikely to experience a shift to normal BMI percentile within a two-year period, as 81.6% of participants were still categorized as having overweight/obesity by the end of follow-up. Although we adjusted for puberty status in our models, the follow-up period was relatively short and may not fully capture the effects of continued growth and maturation on BMI trajectories, underscoring the need for longer-term studies. Our finding is consistent with previous findings that obesity during adolescence often persists for years and that moving from normal BMI to overweight/obesity is common, whereas the opposite is rare [41, 42]. This pattern is critical to long-term cardiometabolic health, as obesity that persists into adulthood substantially increases risk for adverse outcomes [43, 44]. Such findings emphasize the need to understand factors that may affect the trajectories of BMI. Several factors may contribute to this difficulty, including socioeconomic factors and stressors, behavioral factors such as dietary habits and physical activity, and environmental factors such as food availability [45–47].

We found that adolescents with overweight/obesity who had shorter sleep durations were less likely to shift to normal BMI percentile one or two years later. Furthermore, current recommendations suggest that adolescents in this age range sleep 9–12 h per night [48]. In our sample, 65.2% of adolescents slept less than the recommended amount, underscoring the public health relevance of sleep duration as a behavioral factor in body weight trajectories. These findings are consistent with data indicating that sleep duration is an important factor related to body weight regulation. Although prior studies [5, 6] have primarily focused on sleep as a predictor of obesity risk, our findings highlight that sleep also has potential relevance to the likelihood of returning to a normal BMI among adolescents with overweight/obesity. While intervention studies have suggested that improving sleep duration can support weight loss in treatment-seeking individuals [49, 50], there are limited naturalistic, observational studies examining whether sleep duration is associated with the likelihood of returning to a normal BMI [51, 52]. Several possible mechanisms may explain the current findings. For example, short sleep duration among adolescents is associated with changes in appetite-regulating hormones, including increased ghrelin (which promotes hunger) and decreased leptin (which promotes satiety). Insufficient sleep has also been associated with low physical activity [11], changes in glucose metabolism, insulin sensitivity, and cortisol levels, all of which may contribute to weight gain and maintenance [50]. Although we observed a dose-response pattern, the <7 h category did not reach statistical significance, possibly due to the small proportion of participants in this group ( <5%), resulting in limited statistical power.

We found a significant association between short sleep duration and lower likelihood of transitioning to a healthy BMI in both females and males adolescents, and there was no statistical evidence of effect modification by sex (*p* for interaction = 0.598). Our analyses controlled for pubertal status, which is important when examining male and female adolescents since pubertal development rates differ according to sex [53] and influence both sleep and body weight regulation [46, 47]. In complete-case sensitivity analyses, the association in females was not statistically significant, although the effect estimates were directionally consistent with the primary analysis. This attenuation may reflect low statistical power, although the possibility of sex‑specific effects cannot be excluded. Our findings suggest similar associations across sexes, however, larger studies with longer follow-ups are needed to further investigate potential sex differences.

This study found that higher total screen time (continuous or categorical) at baseline was not associated with a lesser likelihood of transitioning to normal BMI. While numerous studies have explored how screen time and sleep contribute to obesity risk [9–11], it remains unclear whether these factors influence the likelihood of returning to a normal BMI among those who already have overweight/obesity. Given the nonsignificant association observed in this study, it is possible that higher screen time does not influence transition from overweight/obese BMI to a healthy level, at least within the short timeframe examined (1–2 years). It is also possible that higher screen time could contribute indirectly to maintenance of overweight/obesity through various pathways, including reducing sleep duration [54, 55]. Previous studies have reported that screen time was associated with bedtime delay and short sleep duration among adolescents [56], which, in turn, may affect BMI transitions following the aforementioned mechanism [50]. Furthermore, our analysis did not find a significant moderation effect of sleep on the screen time–BMI transition association.

The associations of sleep duration and screen time with transition to a normal BMI were not modified by physical activity. Although physical activity is an important factor for weight regulation [57] and is often examined alongside sleep and screen time, in this study it was self-reported using the SAI-Q, which may not fully capture the accumulation of daily physical activity when compared with accelerometery. Future research using objective measures and longer follow-up should explore how sleep and physical activity jointly affect weight trajectories. This joint focus is supported by adolescent intervention studies showing that multicomponent behavior-change lifestyle programs addressing multiple behaviors (e.g., physical activity, diet, sedentary behavior, and sleep) yield modest improvements in weight status [12].

This study had several strengths, including a large, diverse sample of adolescents with overweight/obesity recruited in the U.S., which enhances generalizability. Second, the longitudinal design of the study allowed for the examination of prospective associations, offering insights into factors associated with shifting from overweight/obesity to normal BMI over time among adolescents. Despite these strengths, this study also had some limitations. First, sleep duration was parent-reported, which may introduce reporting bias, as it may not accurately reflect adolescents’ actual sleep duration [58]. Second, the use of BMI categories does not necessarily reflect body fat. Third, adverse childhood experiences (ACEs) were measured only at baseline, which could affect our estimates. Fourth, the follow-up duration was short, and the factors examined were measured at either baseline or Year 1. Given that screen time and sleep duration patterns change during adolescence, using either baseline or Year 1 values as time-invariant exposures may not fully capture their dynamic influence on BMI. Fifth, although we adjusted for numerous theoretically relevant covariates, there may be other salient confounders that were not assessed in this study, such as diet. Finally, due to the observational nature of the study, causality cannot be inferred.

## Conclusion

In this prospective cohort study, short sleep durations were associated with a lower likelihood of moving to a normal BMI among adolescents with overweight/obesity. These findings highlight the importance of addressing short sleep duration as a potential factor influencing adolescents’ BMI. Pediatricians, school health programs, and public health initiatives should integrate sleep education strategy into weight management programs. Schools and families should also play a role by reinforcing healthy sleep habits. Future research should explore additional behavioral and environmental factors that predict weight change across development during adolescence, as well as investigate underlying biological, psychosocial, and behavioral mechanisms.

## Data Availability

Data used in the preparation of this article were obtained from the ABCD Study (https://www.abcdstudy.org), held in the NIMH Data Archive (NDA).
